# Effects of N-Acetylcysteine on Humanin and Endostatin in Rats Exposed to Formaldehyde

**DOI:** 10.7759/cureus.61354

**Published:** 2024-05-30

**Authors:** Feyza Aksu, Ramazan Fazil Akkoc, Ezgi Savur, Celal Çelik

**Affiliations:** 1 Department of Anatomy, Faculty of Medicine, Firat University, Elazig, TUR; 2 College of Medicine, Faculty of Medicine, Firat University, Elazig, TUR

**Keywords:** rat, n-acetylcysteine, humanin, endostatin, exposure to formaldehyde

## Abstract

Introduction: People are constantly exposed to formaldehyde, a volatile and poisonous gas, in indoor environments. In particular, anatomists, pathologists, histologists, and those involved in embalming are exposed to higher amounts of formaldehyde continuously due to their work. This study aimed to investigate the effect of N-acetylcysteine on endostatin and humanin values in male rats exposed to experimental formaldehyde.

Methods: In the study, 28 male Spraque-Dawley rats aged 12-14 weeks (seven animals in each group: control group, formaldehyde group, N-acetylcysteine group, formaldehyde+N-acetylcysteine group) were used. Four weeks later, the animals were sacrificed by decapitation. Following decapitation, endostatin and humanin levels in the serum of rats were studied by the enzyme-linked immunoassay (ELISA) method. In all analyses, p<0.05 was accepted as statistically significant.

Results: Humanin and endostatin values were checked in the serum of rats. When humanin levels were compared between groups, a statistically significant difference was found between the formaldehyde group and both the control group (p<0.05) and the N-acetylcysteine group (p<0.05). In the formaldehyde+N-acetylcysteine group, it was determined that the humanin level was impaired due to formaldehyde exposure, approaching the control group values with the administered N-acetylcysteine.

When the endostatin level was compared between the groups, a statistical significance (p<0.05) was found only between the formaldehyde group and the N-acetylcysteine group. In the formaldehyde+N-acetylcysteine group, it was determined that the endostatin level was impaired due to formaldehyde exposure, approaching the control group values with the administered N-acetylcysteine.

Conclusion: In this study, the effects of N-acetylcysteine on humanin and endostatin on rats exposed to formaldehyde were demonstrated for the first time. Formaldehyde exposure negatively affected humanin and endostatin levels in rat sera. N-acetylcysteine ameliorated the negative effects of formaldehyde, bringing humanin and endostatin levels closer to the healthy control group.

## Introduction

Formaldehyde (FA) is a normal metabolite present in all mammals [1,2]. It is a colorless gas that has a pungent odor, is irritating in its pure form, and is highly soluble in water. FA is a significant chemical with extensive commercial use [1,2]. It is usually stored in the form of a 37% aqueous solution (formalin) and is used as a preservative and sterilizing agent in medical laboratories such as anatomy and histology. Therefore, anatomists, pathologists, histologists, various health personnel, and health department students who frequently have to inhale formaldehyde due to their job may be adversely and severely affected due to exposure to formaldehyde. It has been reported in previous studies that FA causes watery eyes, skin irritation, and damage to lung, kidney, and liver tissues [3-5].

N-acetylcysteine (NAC), which is the precursor of glutathione, is a compound of thiol (containing sulfhydryl). In fact, it has been used as a mucolytic agent for more than 30 years [6]. In addition to its mucolytic effect, NAC is an antioxidant molecule that shows its effect by increasing the intracellular glutathione level. NAC is converted into cysteine in the body. Cysteine is an important amino acid that produces glutathione, which is the body's most powerful antioxidant [7,8]. Another mechanism by which NAC is effective as an antioxidant is that it acts directly on oxidant radicals as a nucleophile at the extracellular level [6]. It has been reported that NAC significantly preserves catalase, SOD, and GSH-Px activities in the model of secondary liver cirrhosis created experimentally in rats. NAC inhibits the oxidative process by elevating antioxidant reserves and protects the liver against this stress [9,10]. Intravenous or oral administration of NAC is also widely used in the treatment of acetaminophen poisoning as it protects the liver [8]. In addition, with its anti-inflammatory and antioxidant effects, NAC mitigates pathological damage in kidney tissue and improves kidney function [11].

Humanin protein is a micropeptide included in the mitochondrial genome [12]. Hashimato et al. first identified humanin by scanning the complementary DNA library obtained from the brain of a person with Alzheimer's disease [13]. Studies have demonstrated the antiapoptotic and neuroprotective effects of humanin on cell survival, metabolism, and inflammation. Since its discovery, it has been identified in a wide variety of tissues such as the testis, colon, hypothalamus, heart, liver, skeletal muscle, kidney, and vascular wall [14-16]. In studies conducted, it has been shown that humanin serum levels decrease with increasing age [15]. Mitochondrial function decreases with age, which leads to an increase in oxidative stress. This situation, which causes oxidative damage to increase with age, has a significant role in cardiovascular diseases (myocardial infarction, hypertension, stroke, etc.) and neurodegenerative diseases (Alzheimer's, Parkinson's, etc.) [17].

Endostatin is a specific endogenous angiogenesis inhibitor that was discovered more than a decade ago [18]. In the light of experimental studies, it has been determined that endostatin can induce apoptosis while inhibiting endothelial cell migration. Therefore, it is well-documented that it prevents a tumor from forming new vessels. Studies have revealed that endostatin produced from collagen XVIII reduces oxidative stress [19]. It has been reported that endostatin plays a role in the progression of the process in kidney and heart diseases where disruption of angiogenesis is seen [20].

FA causes oxidative damage in tissues, while NAC is known to suppress oxidative damage with its antioxidant properties. Endostatin and humanin have also been shown to be effective in oxidative stress. In this study to investigate the healing effects of NAC against FA exposure, it was investigated whether both agents had an effect on endostatin and humanin, and if there was an effect, whether there would be a positive or negative correlation.

## Materials and methods

This study was conducted in the conducted in Firat University, Elazig, Türkiye, between May 1, 2023, to May 1, 2024. The study was approved by the Firat University Animal Experiments Local Ethics Committee (2023/05-07-2023/05-08). In the study, 28 male Spraque-Dawley rats aged 12-14 weeks were included. They were divided into four groups: control group (n=7), FA group (n=7), NAC group (n=7), and FA+NAC group (n=7).

Group-wise intervention

Control Group

The rats in this group were exposed to normal atmospheric air in a 100x50x20 cm bell jar for four weeks, which was the experimental period.

FA Group

The rats in this group were exposed to 10 ppm of FA for eight hours/day for five days a week (except Saturday and Sunday) through inhalation in the 100x50x20 cm bell jar for four weeks [4,5,21].

FA+NAC Group

Throughout the experimental period, rats were exposed to 10 ppm of FA eight hours/day by inhalation in the 100x50x20 cm bell jar and were administered 200 mg/kg/day NAC for five days a week (except Saturday and Sunday) for four weeks through oral gavage [21,22].

NAC Group

The rats in this group were exposed to normal atmospheric air in a 100x50x20 cm bell jar during the experiment period. and NAC was administered by oral gavage at 200 mg/kg/day for four weeks five days a week.

Study of endostatin and humanin levels

After the four weeks of the above intervention, the animals were sacrificed by decapitation. Endostatin levels were studied by using rat endostatin enzyme-linked immunoassay (ELISA) kit (Shanghai Sunred Biological Technology Co., Ltd, Sanghai, China), while humanin levels were studied by using a rat humanin ELISA kit (Shanghai Coon Koon Biotech Co. Ltd., Shanghai, China) as specified in the catalogs of the manufacturers (endostatin catalog no: 201-11-0468, humanin catalog no: CK-BOYO-25638). While the assay range of the endostatin rat ELISA kit was 0.08-20 ng/ml and the minimum measurable level (sensitivity) was 0.071 ng/ml, the assay range of the rat humanin ELISA kit was 20-4000 pg/ml and the minimum measurable level (sensitivity) was 10.0 pg/ml.

In addition, while the intra-assay coefficients of variability (CV) value of the endostatin ELISA kit was <9% and the inter-assay CV value was <11%, the intra-assay CV value of the humanin ELISA kit was <7% and the inter-assay CV value was <10%.

Statistical analysis

For the statistical analysis of the data, IBM SPSS Statistics for Windows, Version 22.0 (Released 2013; IBM Corp., Armonk, New York, United States) was employed. Descriptive statistics were presented as median (minimum-maximum). The Mann-Whitney U test was used to compare two independent groups for quantitative measurements, and the Kruskal-Wallis test was used in the comparison of more than two independent groups. The statistical significance level was set at 0.05 in all tests.

## Results

In FA measurements performed at various hours during the study period, the FA exposure value was measured as 10.20 ± 0.22 ppm. During the four-week study, the hair of the rats in the control group remained white, while yellowing was observed in the hair of the rats in the FA group. In addition, it was observed that the FA group rats exhibited slower movements with frequent nose cleaning, blinking, and licking.

Humanin and endostatin values were checked in the serum of rats. When humanin levels were compared across the groups, a statistically significant difference was found between FA group values and both the control group (p<0.05) and NAC group (p<0.05) values. In the FA+NAC group, it was determined that the humanin level impaired due to exposure to FA converged the control group values as a result of NAC administration, but this improvement was not statistically significant (Table 1).

**Table 1:** Serum endostatin and humanin values ^a^ When the FA group was compared to the control group, p<0.05; ^b^ When the FA group was compared to the NAC group, p<0.05; ^c^ When the FA group was compared to the control group, p<0.05; ^d^ When the FA group was compared to the NAC group, p<0.05 FA: formaldehyde; NAC: N-acetylcysteine

	Control Group	FA Group	NAC Group	FA+NAC Group
Serum endostatin (ng/ml), median (min-max)	5.45 (4.31-5.49)	8.48 (3.31-9.41)^a^	4.82 (4.45-5.92)^b^	5.65 (5.02-6.70)
Serum humanin (pg/ml), median (min-max)	746.4 (713.4-788.4)	675.2 (658.4-691.4)^c^	749.2 (721.8-763.1)^d^	703.2 (689.5-711.2)

When the endostatin levels were compared across the groups, a statistical significance (p<0.05) was found only between the FA group and the NAC group. In the FA+NAC group, it was determined that the endostatin level disrupted due to exposure to FA got closer to the control group values with the administration of NAC, but this improvement was not statistically significant (Table 1).

## Discussion

As indicated in previous studies, yellowing of the hair, loss of appetite, and weakness were observed in the rats as a result of exposure to FA [4,5] .

In an experimental study conducted in the literature, it was reported that as a result of FA applied at different doses, the toxic effect increased as the dose increased [21]. In different experimental studies, the toxic effects of FA have been shown in both liver and kidney tissues [4,5].

In a recent experimental study on rats, NAC was reported to improve gestational diabetes by inhibiting oxidative stress [23]. In another experimental study on rats, it was reported that NAC alleviated apoptosis of bone marrow endothelial cells caused by oxidative stress [24]. In yet another experimental study , it was reported that NAC alleviated oxidative stress and inflammation caused by heat stress [25]. Similar to previous studies, it was determined in the present study that NAC treatment brought humanin and endostatin levels closer to the healthy group against the toxic effects of FA, which has been demonstrated to cause oxidative stress in several studies before [4,5].

In studies conducted in the literature on patients with chronic kidney disease, it has been reported that serum endostatin levels increased compared to the healthy control group [26,27]. Studies have previously been conducted on endostatin levels in different diseases. In a study by Li et al., it was reported that high endostatin levels in cardiovascular diseases may be a marker for disease progression and mortality [20]. In the same study, it was reported that high endostatin levels increased with damage to the kidney tissue, regardless of age, and were indicative of damage [20]. In their study, Salza et al. found that endostatin levels increased significantly in Alzheimer's patients compared to the controls [28]. In this study, serum endostatin levels increased as a result of exposure to FA which had an oxidant effect at toxic doses. This increase decreased again with the administration of NAC, which has an antioxidant effect.

In a study conducted by Zhao et al., it was reported that serum humanin levels decreased significantly in children with inflammatory bowel disease (n=40) compared to healthy controls (n=40) [29]. The study is the first to support the link between inflammation and humanin regulation, and it was reported that serum humanin levels are significantly reduced in children with inflammatory bowel disease [29]. In a study by Yemn et al., it was reported that the decrease in humanin levels in humans, as well as organisms, was associated with some disease parameters [30]. In the study, it was reported that humanin levels, especially in mitochondrial encephalopathy and Alzheimer's patients, decreased significantly compared to the control group. In the same study, it was also reported that low humanin levels were associated with aging and deterioration in the health process [30]. In this study, there was a statistical decrease in serum humanin levels due to exposure to FA, which can have toxic effects on many organs and tissues. NAC treatment, which has an antioxidant effect, increased humanin levels again, which is associated with longevity and health. However, this difference was not statistically significant.

Limitations of the study

This study was conducted on a small sample of rats (n=28) and was limited to serum humanin and endostatin ELISA results. Tissue ELISA values and immunohistochemistry could not be studied. More comprehensive studies are needed on this subject.

## Conclusions

In this study, the effect of NAC administration on serum endostatin and humanin levels in exposure to FA was demonstrated for the first time. Exposure to FA leads to a toxic effect on serum endostatin and humanin levels. NAC, which is applied as a treatment, has a curative effect on endostatin and humanin levels.
